# Hypercalcemia as a rare manifestation of immune reconstitution inflammatory syndrome (IRIS) in a person living with Human Immunodeficiency Virus (HIV) with disseminated nontuberculous mycobacteriosis

**DOI:** 10.1007/s15010-024-02228-7

**Published:** 2024-03-23

**Authors:** Maximilian Webendoerfer, Margarethe Konik, Markus Zettler, Johannes Wienker, Josefine Rawitzer, Stefan Esser, Jan Kehrmann, Ken Herrmann, Hans Christian Reinhardt, Oliver Witzke, Sebastian Dolff

**Affiliations:** 1 Department of Medical Oncology, West German Cancer Center, University Hospital Essen, University of Duisburg-Essen, Hufelandstraße 55, 45147 Essen, Germany; 2 Department of Infectious Diseases, West German Center of Infectious Diseases, Essen University Hospital, University of Duisburg-Essen, Hufelandstraße 55, 45147 Essen, Germany; 3 Department of Pneumology, University Hospital Essen - Ruhrlandklinik, University of Duisburg-Essen, Tüschener Weg 40, 45239 Essen, Germany; 4 Institute for Pathology and Neuropathology, University Hospital Essen, University of Duisburg-Essen, Hufelandstraße 55, 45147 Essen, Germany; 5 Department of Dermatology and Venereology, HIV Outpatient Clinic, University Hospital Essen, University of Duisburg-Essen, Hufelandstraße 55, 45147 Essen, Germany; 6 Institute of Medical Microbiology, University Hospital Essen, University of Duisburg-Essen, Hufelandstraße 55, 45147 Essen, Germany; 7 Department of Nuclear Medicine, University Hospital Essen, University of Duisburg-Essen, Hufelandstraße 55, 45147 Essen, Germany; 8 Department of Hematology and Stem Cell Transplantation, University Hospital Essen, University of Duisburg-Essen, Hufelandstraße 55, 45147 Essen, Germany

**Keywords:** Hypercalcemia, HIV, IRIS, DLBCL, *Mycobacterium avium-intracellulare*, Acute kidney damage

## Abstract

**Introduction:**

Granulomatosis due to immune reconstitution inflammatory syndrome (IRIS) and disseminated *Mycobacterium avium-intracellulare* (*M. avium*) infection may trigger hypercalcemia. Here, we report a rare case of hypercalcemia and acute kidney damage related to IRIS in a person living with Human Immunodeficiency Virus (HIV).

**Case presentation:**

A 39-year-old male person living with HIV presented with muscle weakness and unwanted weight loss of 8 kg within the last 2 weeks. Laboratory findings included serum hypercalcemia of 3.27 mmol/mL associated with elevated calcitriol and acute kidney damage. Since the first diagnosis of HIV and concomitant disseminated *M. avium* infection, the patient received antiretroviral therapy (ART), rifabutin, clarithromycin, and ethambutol. ^18^Fluoro-D-glucose positron emission computed tomography (^18^FDG-PET/CT) showed progressive multilocular lymphadenopathy. Biopsy specimen from the duodenum as well as retroperitoneal and mediastinal lymph nodes revealed granulomatous inflammation consistent with IRIS. Treatment with forced diuresis, bisphosphonates, and calcitonin normalized serum calcium and kidney function recovered.

**Conclusion:**

Hypercalcemia due to IRIS is a rare differential diagnosis in persons living with HIV and may lead to acute kidney damage, despite sufficient ART and antimycobacterial treatment.

## Case presentation

A 39-year-old male person living with HIV presented to the emergency department with progressive muscle weakness and unintended weight loss of 8 kg within the last 2 weeks. He reported watery stool twice per day for 3 months. There was no fever or other clinical signs of acute infection. For further diagnostic assessment and clinical surveillance, the patient was admitted to our center for internal medicine.

**Fig. 1:**
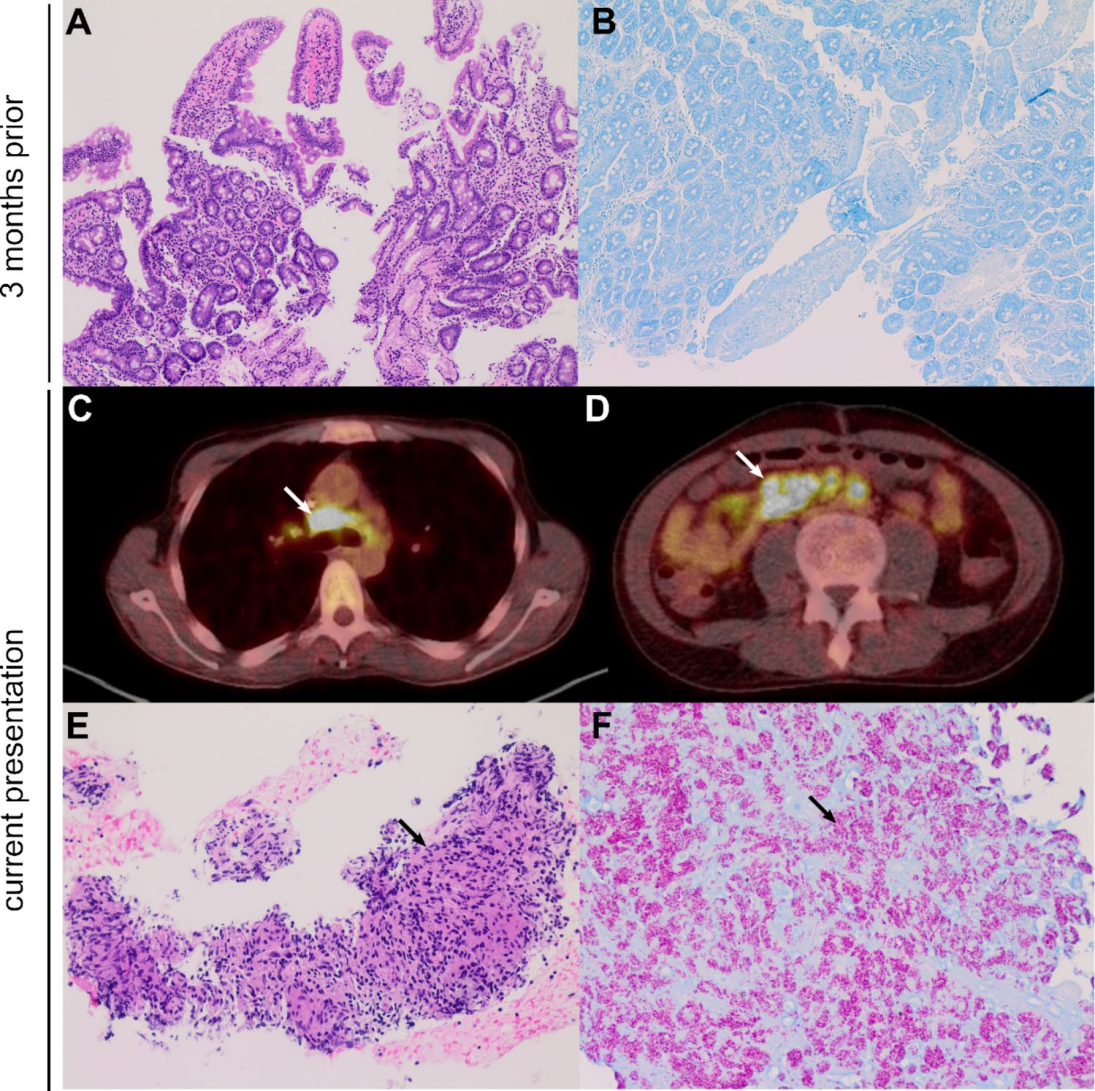
Duodenal biopsy at the time of HIV diagnosis (**A**, **B**) and granuloma formation after 3 months of antimycobacterial and ART (**C**–**F**). Hematoxylin–eosin staining (HE) (**A**; 100×) and Ziehl–Neelsen staining (**B**; 100×) of a duodenal biopsy at the time of initial HIV and *M. avium* infection diagnosis with discrete inflammation and no acid-fast bacilli. Thoracic (**C**) and abdominal (**D**) images from a^18^FDG-PET/CT scan at current presentation showing progressive multilocular lymphadenopathy (arrow). HE staining (**E**; 200×) and Ziehl–Neelsen staining (**F**; 100×) showing mediastinal lymph node biopsy samples with granuloma (arrow) and abundant acid-fast bacilli (arrow)

The initial diagnosis of HIV infection was made in our center 3 months prior to this presentation. At initial staging of the disease, the patient had a high viral load of 1.120.000 HIV copies/mL and an absolute CD4^+^ T-helper cell count of 38/µL. Screening for opportunistic infections and acquired immunodeficiency syndrome (AIDS)-defining conditions revealed a disseminated *M. avium* infection*,* cytomegalovirus infection with a viral load of 9.500 international units/mL, and wasting syndrome consistent with AIDS according to the WHO clinical staging system [1]. Computer tomography (CT) of the chest showed multiple slightly enlarged mediastinal and axillary lymph nodes, which were attributed to HIV infection. The patient’s medical history was notable for diffuse large B cell lymphoma (DLBCL) with primary gastric manifestation (Ann Arbor Stage I_E_) 15 months prior to this presentation and he had been treated with four cycles of chemoimmunotherapy according to R-CHOP protocol. No HIV test was performed at the time of diagnosis of DLBCL at an external hospital. The first follow-up staging 9 months prior to this presentation was consistent with complete remission of the disease. At the time of initial HIV and *M. avium* infection diagnosis, esophagogastroduodenoscopy (EGD) showed no sign of recurrence of lymphoma and duodenal biopsies ruled out granulomatosis (Fig. 1A, B).

Up to this presentation, the patient took a daily single tablet ART regimen of abacavir/lamivudine/dolutegravir (Triumeq) and a combined, antibiotic regimen of rifabutin, ethambutol, and clarithromycin. He reported consistent intake of ART, as well as antimycobacterial therapy. He took no thiazide diuretic or Vitamin D supplement.

Upon physical examination, the patient exhibited signs of cachexia and hypovolemia, but no clinical symptoms of acute infection. Initial laboratory values were notable for moderate hypercalcemia of 3.27 mmol/L, increased serum creatinine of 1.77 mg/dL, and moderately increased C-reactive protein of 3.8 mg/dL. Serum albumin was moderately decreased to 3.0 g/dL. LDH was normal. Abdominal ultrasound excluded a post-renal cause of acute kidney injury and revealed splenomegaly with an inhomogeneous splenic parenchyma. Forced diuresis with balanced electrolyte solution and furosemide was started. Despite this therapy, hypercalcemia, as well as acute kidney injury aggravated to a serum creatinine of 3.64 mg/dL.

Further laboratory work-up revealed a decreased HI viremia (170 copies/mL) and an increased absolute CD4^+^ T-helper cell count (152/µL) as compared to the time of initial HIV diagnosis, consistent with the patient’s reported therapy adherence. B cells were depleted with an absolute count of 1/µL following rituximab therapy 12 months before the current presentation. Repeated blood cultures were negative. PTH was adequately suppressed.

The patient’s refractory hypercalcemia correlated with an increased calcitriol of 118 pg/mL (reference range—79 pg/mL). An ^18^FDG-PET/CT scan showed multilocular lymphadenopathy including cervical, mediastinal, abdominal, as well as retroperitoneal lymph nodes (Fig. 1C, D). As we suspected recurrence of lymphoma, no steroids were given at that time. Biopsy specimen from mediastinal lymph nodes using endobronchial ultrasound-guided needle biopsy, retroperitoneal lymph nodes using CT-guided percutaneous biopsy, as well as duodenal biopsy using EGD were obtained to rule out recurrence of lymphoma. All biopsies showed granulomatous inflammation (Fig. 1E). Microscopy and PCR confirmed presence of *M. avium* in mediastinal lymph nodes (Fig. 1F). Interestingly, the patient’s duodenal biopsy showed no sign of granulomatosis following initial detection of *M. avium* in blood cultures 3 months prior to this presentation (Fig. 1A).

After initiation of calcitonin and bisphosphonates, the patient’s serum calcium levels normalized rapidly, and kidney function recovered. Mycobacterial cultures from mediastinal lymph node biopsies confirmed presence of *M. avium*. There was no sign of macrolide resistance of the *M. avium* isolate using molecular testing. The initial ART and antimycobacterial therapy regimen were continued. In a follow-up visit 1 month later, the patient presented in good general health and reported no acute symptoms. Serum calcium levels were normal. Calcitriol was still increased, albeit reduced to 89 pg/mL. The patient’s kidney function recovered.

## Discussion

Hypercalcemia is a common electrolyte disorder, which may be linked to malignant disease or primary hyperparathyroidism in approximately 90% of cases [2]. Depending on the patient population, causes of hypercalcemia vary. As compared to the general population, persons living with HIV are more likely to develop hypercalcemia (3.2% vs. 1 to 2%, [2, 3]). Solid and hematologic malignancies as well as opportunistic infections, including tuberculosis, nontuberculous mycobacterial disease, *pneumocystis jirovecii* pneumonia, and cryptococcosis, account for most cases of hypercalcemia in persons living with HIV [3]. The clinical symptoms range from asymptomatic presentation in mild hypercalcemia to nausea, vomiting, obstipation, QT time shortening, and muscle weakness in rapid onset and severe hypercalcemia [2]. Regarding kidney dysfunction, hypercalcemia is associated with nephrogenic diabetes insipidus leading to polyuria, polydipsia, and extracellular volume contraction. This, alongside hypercalcemia-mediated arterial vasoconstriction, is hypothesized to facilitate acute kidney damage [4].

In our patient, a history of DLBCL, as well as disseminated infection with *M. avium* presented a diagnostic challenge due to worsening hypercalcemia and subsequent kidney damage. Relapses in patients with DLBCL treated with R-CHOP as first-line therapy affect 30–50% of patients and most relapses occur within the first 2 years following treatment [5]. Relapses of DLBCL and subsequent paraneoplastic hypercalcemia are less common in lower disease stages. Hypercalcemia due to IRIS and disseminated mycobacteriosis is even more rare and has only been recognized in case reports and small case series [3, 6, 7]. In our patient, hypercalcemia developed due to elevated calcitriol. Nonetheless, recurrence of DLBCL, as well as disseminated granulomatosis due to *M. avium* infection may facilitate hypercalcemia because of ectopic production of calcitriol [2, 3, 8]. Current guidelines recommend glucocorticoids in the context of calcitriol-mediated hypercalcemia [9]. However, pre-biopsy glucocorticoid treatment may obscure the histopathological diagnosis of recurrent lymphoma, as relapsing DLBCL may present with differing histology compared to the initial diagnosis [5]. In addition, treatment with glucocorticoids renders patients susceptible to mycobacterial infections and may increase mycobacterial survival through inhibition of phagocytosis and autophagy [10]. Our patient developed a severe complication of *M. avium* infection, despite sufficient antimycobacterial and antiretroviral therapy. Neither during first diagnosis of *M. avium* infection nor at the current presentation 3 months later, we detected a resistance to combination antimycobacterial therapy. Therefore, treatment failure of antimycobacterial therapy is unlikely to explain the patient’s hypercalcemia. We hypothesize the patient developed granulomatous inflammation due to paradoxical IRIS. The International Network for the Study of HIV-associated IRIS (INSHI) differentiates paradoxical IRIS from ART-associated IRIS and unmasking tuberculosis-associated IRIS [11]. Paradoxical IRIS is diagnosed in patients with tuberculous mycobacteriosis prior to initiation of ART presenting with at least one diagnostic mayor criterion or two minor criteria within 3 months of ART initiation and after excluding other explanations for these findings [11]. Extending the definition of paradoxical IRIS to nontuberculous mycobacteriosis, our patient fulfilled progressive multilocular lymphadenopathy as mayor criterium and weight loss and splenomegaly as minor criteria for the diagnosis of paradoxical IRIS. Our patient had several risk factors predisposing for IRIS, which included a low absolute CD4^+^ T cell count at the time of initiating ART, a rapid increase in CD4^+^ T cells and HIV RNA viral suppression following initiation of ART, as well as a pre-existing opportunistic infection with *M. avium* with a high antigenic burden [12]. Furthermore, our patient received Dolutegravir as an integrase inhibitor. Dolutegravir has been associated with a higher risk of IRIS in a retrospective analysis [13]; nonetheless, a recent meta-analysis did not confirm this finding [14]. While nontuberculous mycobacteriosis is common in persons living with HIV, few cases of hypercalcemia due to *M. avium* infection are described and pathomechanisms are heterogeneous [3, 6, 7, 15]. Elevated calcitriol has been reported [7, 15], others have detected only normal levels of calcitriol [6]. In our patient, granulomatosis in the duodenum developed only after initiation of antiretroviral therapy and T cells reconstituted. This clinical observation mirrors preclinical in vivo data of a mouse model of granulomatosis in which CD4^+^ T-cell-depleted humanized mice were unable to form granulomas upon mycobacterial infection [16]. As the patient received chemoimmunotherapy including rituximab, prior to HIV diagnosis, a combined deficiency of T and B cell function may contribute to the delayed formation of granuloma and IRIS-related hypercalcemia. In our patient, the diagnosis of DLBCL preceded the diagnosis of HIV infection. To the best of our knowledge, the patient was not tested for HIV at the time of diagnosis of DLBCL at an external hospital. An HIV infection may have occurred after the diagnosis of DLBC. Still, this may have been a missed chance for an earlier detection of HIV infection, especially since current guidelines recommend testing for HIV and viral Hepatitis at the time of lymphoma diagnosis [17].

## Conclusion

Hypercalcemia is a clinical condition with potentially severe outcome. Causes of hypercalcemia vary in different patient populations and include hematological and solid malignancies, hyperparathyroidism, as well as infections. Persons living with HIV are prone to opportunistic infections, as well as malignant disease; hence, the differential diagnosis for hypercalcemia should be broad. Granuloma formation due to IRIS may facilitate hypercalcemia despite sufficient antimycobacterial therapy and ART in persons living with HIV and disseminated *M. avium* infection.

## Data Availability

No datasets were generated or analysed during the current study.

## References

[1] World Health Organization. Consolidated guidelines on the use of antiretroviral drugs for treating and preventing HIV infection: recommendations for a public health approach. WHOint. 2016; https://iris.who.int/handle/10665/208825. Accessed 23 Feb 2024. 27466667

[2] Walker MD, Shane E. Hypercalcemia. JAMA. 2022;328:1624. 10.1001/jama.2022.18331. 36282253 10.1001/jama.2022.18331

[3] Nongnuch A, Petcharut J, Suksuwan W, Davenport A, Phuphuakrat A. Causes of hypercalcemia in people living with HIV in the HAART era. HIV Res Clin Pract. 2020;21:115–20. 10.1080/25787489.2020.1836900. 33076771 10.1080/25787489.2020.1836900

[4] Thongprayoon C, Cheungpasitporn W, Mao MA, Sakhuja A, Erickson SB. Admission calcium levels and risk of acute kidney injury in hospitalised patients. Int J Clin Pract. 2018;72: e13057. 10.1111/ijcp.13057. 29314467 10.1111/ijcp.13057

[5] Maurer MJ, Ghesquières H, Jais JP, Witzig TE, Haioun C, Thompson CA, et al. Event-free survival at 24 months is a robust end point for disease-related outcome in diffuse large B-cell lymphoma treated with immunochemotherapy. J Clin Oncol. 2014;32:1066–73. 10.1200/jco.2013.51.5866. 24550425 10.1200/jco.2013.51.5866 PMC3965261

[6] Chatterjee T, Reddy YPS, Kandula M. Mycobacterium avium complex: an unusual cause of hypercalcemia. IDCases. 2021;26: e01317. 10.1016/j.idcr.2021.e01317. 34786338 10.1016/j.idcr.2021.e01317 PMC8577481

[7] Awasty SS, Jafri S, Manzoor S, Yaqub A. Hypercalcemia secondary to immune reconstitution inflammatory syndrome in an HIV-infected individual with Mycobacterium avium complex. Cureus. 2021. 10.7759/cureus.18174. 34703699 10.7759/cureus.18174 PMC8530549

[8] Shallis RM, Rome RS, Reagan JL. Mechanisms of hypercalcemia in non-Hodgkin lymphoma and associated outcomes: a retrospective review. Clin Lymphoma Myeloma Leuk. 2018;18:e123–9. 10.1016/j.clml.2017.12.006. 29361495 10.1016/j.clml.2017.12.006

[9] Fuleihan GEH, Clines GA, Hu MI, Marcocci C, Murad MH, Piggott T, et al. Treatment of hypercalcemia of malignancy in adults: an endocrine society clinical practice guideline. J Clin Endocrinol Metab. 2022. 10.1210/clinem/dgac621. 10.1210/clinem/dgac621 36545746

[10] Wang J, Wang R, Wang H, Yang X, Yang J, Xiong W, et al. Glucocorticoids suppress antimicrobial autophagy and nitric oxide production and facilitate mycobacterial survival in macrophages. Sci Rep. 2017. 10.1038/s41598-017-01174-9. 28428627 10.1038/s41598-017-01174-9 PMC5430514

[11] Meintjes G, Lawn SD, Scano F, Maartens G, French MA, Worodria W, Elliott JH, Murdoch D, Wilkinson RJ, Seyler C, John L, van der Loeff MS, Reiss P, Lynen L, Janoff EN, Gilks C, Colebunders R, International Network for the Study of HIV-Associated IRIS. Tuberculosis-associated immune reconstitution inflammatory syndrome: case definitions for use in resource-limited settings. Lancet Infect Dis. 2008. 10.1016/S1473-3099(08)70184-1. 18652998 10.1016/S1473-3099(08)70184-1 PMC2804035

[12] Murdoch DM, Venter WD, Van Rie A, Feldman C. Immune reconstitution inflammatory syndrome (IRIS): review of common infectious manifestations and treatment options. AIDS Res Ther. 2007;4:9. 10.1186/1742-6405-4-9. 17488505 10.1186/1742-6405-4-9 PMC1871602

[13] Wijting IEA, Wit FWNM, Rokx C, Leyten EMS, Lowe SH, Brinkman K, et al. Immune reconstitution inflammatory syndrome in HIV infected late presenters starting integrase inhibitor containing antiretroviral therapy. EClinicalMedicine. 2019;13: 100210. 10.1016/j.eclinm.2019.11.003. 10.1016/j.eclinm.2019.11.003 PMC6933261 31891143

[14] Zhao Y, Hohlfeld A, Namale P, Meintjes G, Maartens G, Engel ME. Risk of immune reconstitution inflammatory syndrome with integrase inhibitors versus other classes of antiretrovirals: a systematic review and meta-analysis of randomized trials. J Acquir Immune Defic Syndr. 2022;90:232–9. 10.1097/qai.0000000000002937. 35175970 10.1097/qai.0000000000002937 PMC7612870

[15] Tsao YT, Lee SW, Hsu JC, Ho FM, Wang WJ. Surviving a crisis of HIV-associated immune reconstitution syndrome. Am J Emerg Med. 2012;30:1661.e5-7. 10.1016/j.ajem.2011.09.003. 22033392 10.1016/j.ajem.2011.09.003

[16] Heuts F, Gavier-Widén D, Carow B, Juarez J, Wigzell H, Rottenberg ME. CD4 + cell-dependent granuloma formation in humanized mice infected with mycobacteria. Proc Natl Acad Sci U S A. 2013;110:6482–7. 10.1073/pnas.1219985110. 23559373 10.1073/pnas.1219985110 PMC3631626

[17] Tilly H, Gomes da Silva M, Vitolo U, Jack A, Meignan M, Lopez-Guillermo A, Walewski J, André M, Johnson PW, Pfreundschuh M, Ladetto M. Diffuse large B-cell lymphoma (DLBCL): ESMO clinical practice guidelines for diagnosis, treatment and follow-up. Ann Oncol. 2015. 10.1093/annonc/mdv304. 26314773 10.1093/annonc/mdv304

